# Primary orbital pleomorphic liposarcoma in a child: A case report

**DOI:** 10.1016/j.ajoc.2022.101285

**Published:** 2022-01-20

**Authors:** Trakanta Wannapanich, Paitoon Pratipanawat

**Affiliations:** Faculty of Medicine, Khon Kaen University, Thailand

**Keywords:** Primary orbital liposarcoma, Pleomorphic liposarcoma, Pediatric sarcoma

## Abstract

**Purpose:**

To report a rare case of primary orbital pleomorphic liposarcoma and present a relevant literature review.

**Observations:**

An 11-year-old boy presented with an enlarging, painless lower right eyelid mass that was noted 4 months ago. Imaging revealed a 3.2 × 2.1 × 3.7-cm-sized well-circumscribed lobulated mass. Biopsy revealed a pleomorphic spindle cell neoplasm that was consistent with a pleomorphic liposarcoma. A systemic evaluation found no evidence of distant metastasis. Despite four chemotherapy cycles, the mass size increased at follow-up. Total orbital exenteration was performed 1 year after confirming a diagnosis of pleomorphic liposarcoma. At the 6-month follow-up, the patient was alive with no signs of recurrence.

**Conclusions:**

Pediatric pleomorphic liposarcoma is a very rare entity. Its definite diagnosis relies on histopathological results. In the absence of systemic metastasis, total orbital exenteration is the optimal approach for local control.

## 1 Introduction

Liposarcoma is a common soft tissue tumor in adults but a very rare entity in children, accounting for <3% of all pediatric sarcomas.^1^ According to the 2020 World Health Organization soft tissue and bone tumors classification, malignant adipocytic tumors are histologically classified as well-differentiated, myxoid, pleomorphic, myxoid pleomorphic, and dedifferentiated liposarcomas.^2^ Well-differentiated liposarcoma is the most common subtype in adults,^3^ whereas the myxoid subtype is the most common in children.^4^

Pleomorphic liposarcoma is the rarest subtype (5% of all liposarcomas).^3^ The most common primary sites are the extremities, followed by the internal trunk, and the prognosis is generally unfavorable, with high rates of local recurrences. More than one-third of patients who undergo complete resection develop distant metastasis.^5^

The orbit is an extremely rare primary site for liposarcomas and even rarer for pleomorphic subtypes. Only seven case reports of primary orbital pleomorphic liposarcoma have been found in the literature since 1990, and only two of them were in patients aged <20 years.^6^, ^7^, ^8^, ^9^, ^10^, ^11^, ^12^ This report presents a rare case of a primary orbital pleomorphic liposarcoma in an 11-year-old boy.

## 2 Case report

An 11-year-old Asian boy with an enlarging lower right eyelid mass was referred to our hospital for further evaluation and complete tumor removal. The child had homozygous hemoglobin E disease, with no anemic symptoms, as an underlying disease. He denied any significant past ocular history or family history of malignancy at a young age. Four months ago, he first noticed this painless mass of approximately 1 cm. Two months after the mass had developed, the child underwent contrast-enhanced orbital magnetic resonance imaging (MRI), which revealed a 3.2× 2.1 × 3.7-cm-sized well-circumscribed lobulated mass on the lower right eyelid, abutting on the right inferior rectus muscle and causing superior right eye globe displacement.

**Fig. 1:**
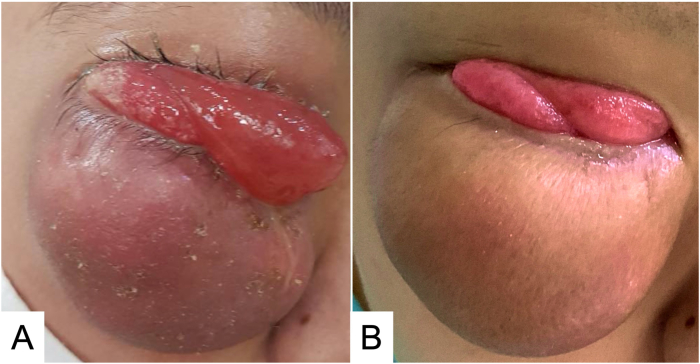
External appearance of the patient's right orbit. (A) At the first presentation to our hospital. (B) After 4 cycles of chemotherapy.

**Fig. 2:**
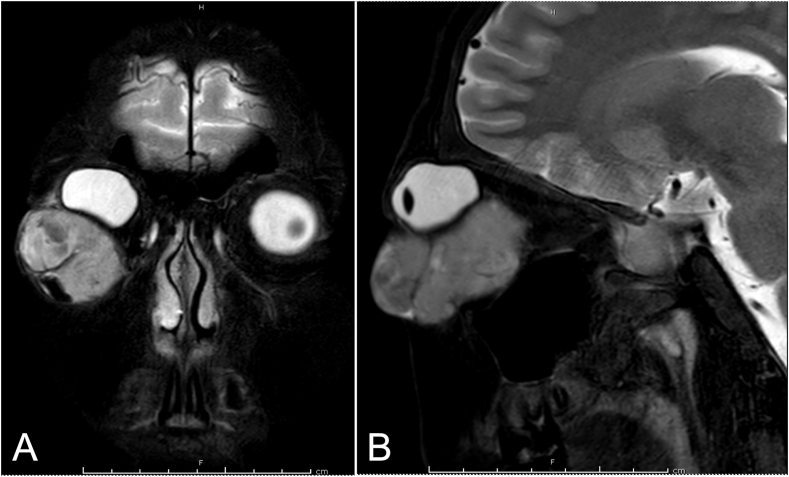
Orbital T2-weighted MRI with fat suppression of the patient at 3 months after the first presentation. (A) Coronal view. (B) Sagittal view.

On physical examination, his best-corrected visual acuity was 2/60 OD and 6/12 OS, with normal pupil reaction. External examination showed a 5-cm- painless, firm lower right eyelid mass. The mass caused superior globe displacement, complete ptosis, marked conjunctival chemosis, and limited extraocular muscle movement, with the greatest restriction on the inferior directions (Fig. 1A). The left eye examination was unremarkable, with full motility in all directions. Clinical differential diagnosis included orbital tumor not otherwise specified and vascular malformations, including venous lymphatic and cavernous venous malformations. Follow-up MRI after 1 month revealed an increase in the solid mass size to approximately 4.9 × 3.9 × 3.2 cm, with posterior extension to the anterior right orbit extraconal space (Fig. 2). Incisional biopsy revealed a pleomorphic spindle cell neoplasm with hyperchromatic nuclei, high-N/C ratio, coarse chromatin and some cells showed lipoblast-like features. The results were consistent with a pleomorphic liposarcoma. A rare mitosis was noted (0–9 mitoses per 10 high-power field). Immunohistochemical examinations were negative for AE1/AE3, EMA, S100, and HMB45. The patient was referred to the pediatric oncology unit. Further evaluations including brain imaging, chest computed tomography, and bone scan found no evidence of distant metastases.

**Fig. 3:**
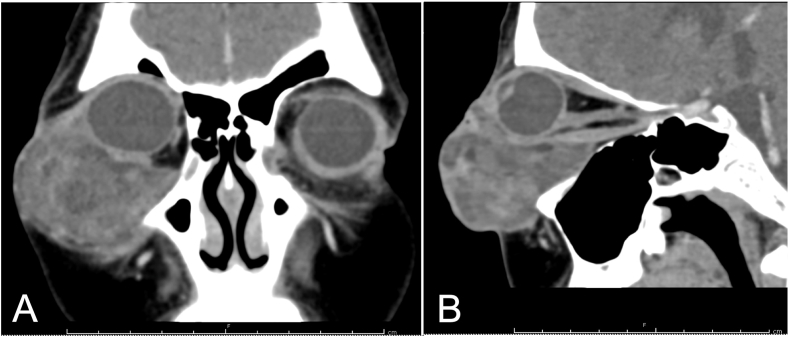
Orbital CT scan of the patient after the 4th cycle of chemotherapy. (A) Coronal view without contrast. (B) Sagittal view without contrast.

Neoadjuvant chemotherapy (protocol ThaiPOG-NRMS-1801 for non-rhabdomyosarcoma, Arm D), including ifosfamide and doxorubicin, was administered as four cycles in total (3 weeks apart). The mass slightly increased in size at the follow-up examination, and a trace relative afferent pupillary defect of the right eye was noted (Fig. 1B). Orbital computed tomography revealed that the heterogenous enhancing mass increased in size, measuring approximately 5.0 × 6.0 × 5.0 cm. The tumor caused pressure that pushed the right eye globe superiorly and the superior maxillary sinus inferiorly. The extraconal and intraconal fats were also involved in the tumor (Fig. 3). Total exenteration of the right orbit was performed 13 weeks after chemotherapy was initiated. The socket was left to granulate. Pathological results confirmed the pleomorphic liposarcoma diagnosis, and the tumor measured approximately 5.5 × 5.0 × 4.0 cm (Supplementary Figs. S1 and S2). All margins except the lateral ones were uninvolved with the tumor (anterior, posterior, and inferior <1 mm, superior 20 mm, medial 2 mm). The patient received postoperative adjuvant chemotherapy, following the same protocol as in the preoperative period. Intensity-modulated radiation therapy (IMRT) was administered with 200 cGy per fraction for 33 fractions (a total dose of 6600 cGy) during a 6-week period to counter possible microscopic residual tumor tissues. No significant change in the patient's anemic profile was noted after treatment completion. No signs of recurrence were noted at the 6-month post-exenteration follow-up.

## 3 Discussion

Liposarcoma of the head and neck region is a rare entity. The pleomorphic subtype is a high-grade undifferentiated sarcoma with a variable number of pleomorphic lipoblasts and has the most aggressive course, with a 1-year survival rate of approximately 45%.^5^ This tumor was reported to rarely metastasize but could be locally invasive. The most common metastatic site was the lungs, followed by the liver and skeleton.^5^ We found only seven case reports on primary orbital pleomorphic liposarcomas after reviewing the literature since 1990 (Table 1).

**Table 1:** Literature reports of primary orbital pleomorphic liposarcomas.

Author	Age/Sex	Symptoms/Duration	Location	Size	Treatment	Follow-up
McNab and Moseley^6^	52/M	Blurred vision/7 months	Intraconal, abutting the medial rectus and upper surface of the optic nerve	2 cm	Exenteration	12 months NSR
Cai et al.^7^	51/M	Diplopia, proptosis/several months	Retrobulbar and inferior involving lower eyelid	4 cm	Exenteration	65 months NSR
Monteiro^8^	75/M	Diplopia, proptosis/3 months	Medial rectus muscle	NA	Exenteration, post-op RT	6 years NSR
Madge et al.^9^	19/M	Proptosis/5 months	Extraconal, superomedial quadrant of the orbit	NA	Local resection, CMT, exenteration, post-op RT	9.5 years NSR
Rudzinski et al.^10^	8/M	Swollen eye/3 months	Extraconal, anterior to superior rectus muscle insertion	NA	Local resection, CMT, post-op RT	Death after 2 years due to metastasis
Doyle et al.^11^	62/F	Diplopia, proptosis/5 months	Retrobulbar, invading the paranasal sinus	5 cm	Local resection	Lost to follow-up after 2 years
Jang et al.^12^	59/M	Proptosis/NA	Retrobulbar	NA	Exenteration, post-op RT	Death after 1 year due to metastasis

CMT, chemotherapy; F, female; M, male; NA, information not available; NSR, no sign of recurrence; RT, radiotherapy.

The tumor was surgically resected in all of the cases, either via local resection or exenteration. Two patients died, whereas the others showed no sign of recurrence or were lost to follow-up.^6^, ^7^, ^8^, ^9^, ^10^, ^11^, ^12^

Liposarcoma is extremely uncommon in children. The most common primary site in adults is the extremities. Madge et al. reported on a 19-year-old Caucasian man with primary orbital pleomorphic liposarcoma.^9^ The patient was treated with combined chemotherapy and local resection and underwent exenteration 5 years after the initial presentation. As multiple recurrences were found, the patient was treated with combined chemotherapy, radiotherapy, and multiple tumor resections before the disease was controlled. Rudzinski et al. reported on a primary orbital pleomorphic liposarcoma in an 8-year-old boy who presented with a swollen left eye for 3 months.^10^ Initially, there was no evidence of metastasis; however, he later developed metastatic disease. Despite aggressive treatments, he died 2 years after the initial symptoms.

The treatment option for liposarcomas is primarily surgical resection, and it is generally recommended to excise at least 2 cm of negative surgical margins in cases of soft tissue sarcomas.^13^ However, the possibility of achieving this wide margin is more difficult in the head and neck region than in the rest of the body. Only 2–5% of head and neck liposarcoma cases were treated with adjuvant radiotherapy, which is a rate lower than that observed for other body regions. This was explained by its inconclusive efficacy and overall survival benefits.^14^ In our case, postoperative radiotherapy was performed to improve local tumor control because there was a positive surgical margin. Chemotherapy might provide an additional benefit for localized resectable soft tissue sarcomas, as was demonstrated in a meta-analysis.^15^ The combination of doxorubicin and ifosfamide is the preferred treatment; however, this treatment must be weighed against associated toxicities. Our patient tolerated the systemic chemotherapy well, with no documented severe adverse events.

## 4 Conclusions

We described the clinical features of an 11-year-old boy with primary orbital pleomorphic liposarcoma, a rare entity. Diagnosis primarily depended on histopathological findings obtained via biopsy. In the absence of systemic metastasis, total exenteration of the orbit with adequate surgical margin seemed to be the best treatment option. Adjunctive chemotherapy or radiotherapy might improve local control, although the decision should be weighed against the risk. Clinicians should consider liposarcoma as a differential diagnosis of orbital masses in children.

## Author statement

**Trakanta Wannapanich:** Conceptualization, Methodology, Validation, Writing - Original Draft, Writing - Review & Editing, Visualization.

**Paitoon Pratipanawat:** Validation, Writing - Review & Editing, Supervision, Project administration, Funding acquisition.

## Patient consent

Both patient and patient's legal guardian consented to publication of the case in writing. The patient and his parents have given their permission to publish their clinical data and images.

## Funding

Faculty of Medicine, Khon Kaen University, Thailand.

## Declaration of competing interest

The authors declare that there are no conflict of interests of this paper.

## Appendix A Supplementary data

figs1 figs1

figs2 figs2
