# Supplementary figures and images for: Primary orbital pleomorphic liposarcoma in a child: A case report
Source: Am J Ophthalmol Case Rep. 2022 Jan 20;25:101285. doi: 10.1016/j.ajoc.2022.101285 (PMC8818483; doi:10.1016/j.ajoc.2022.101285)

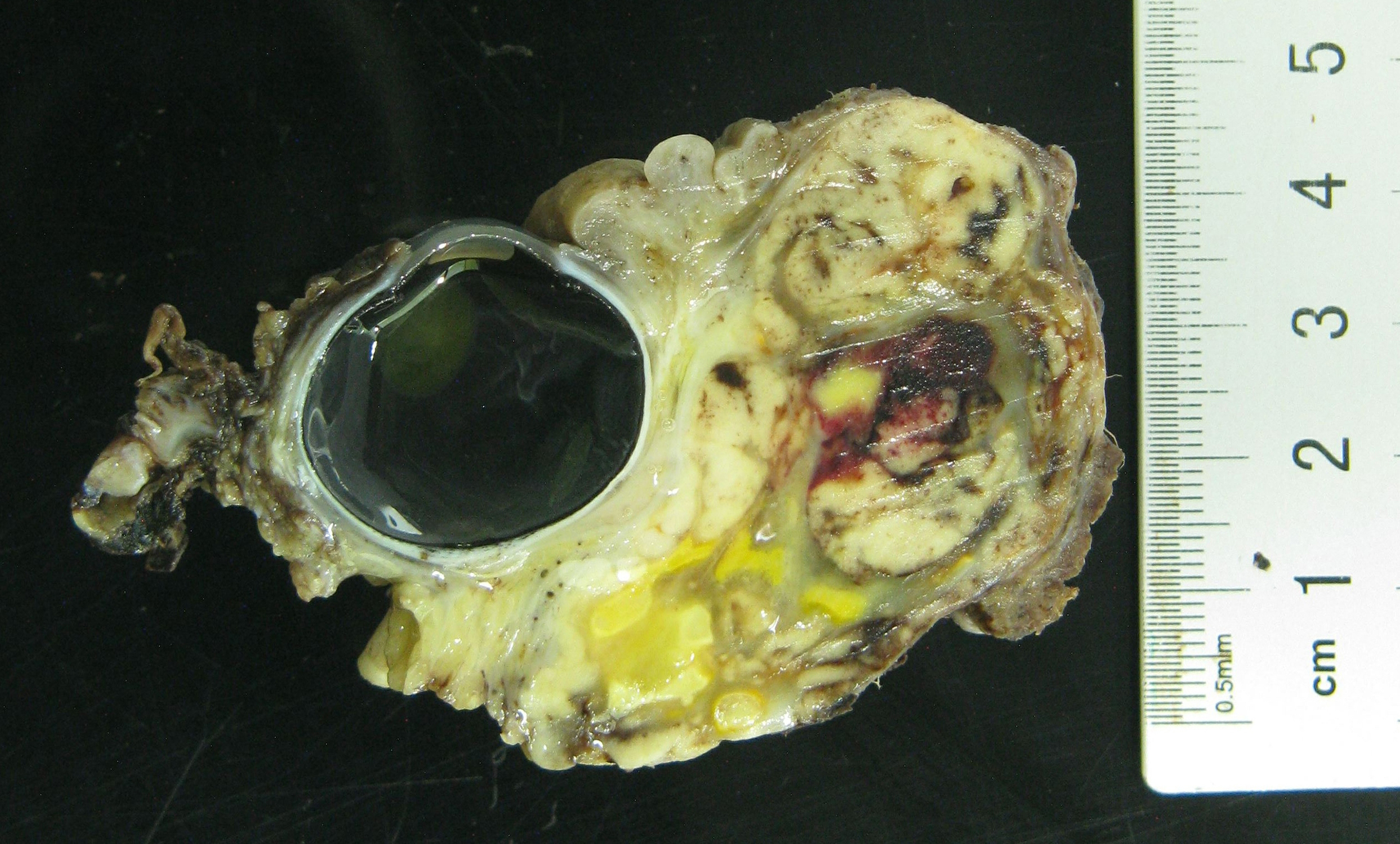

Supplement: figs1 [file mmcfigs1.jpg]

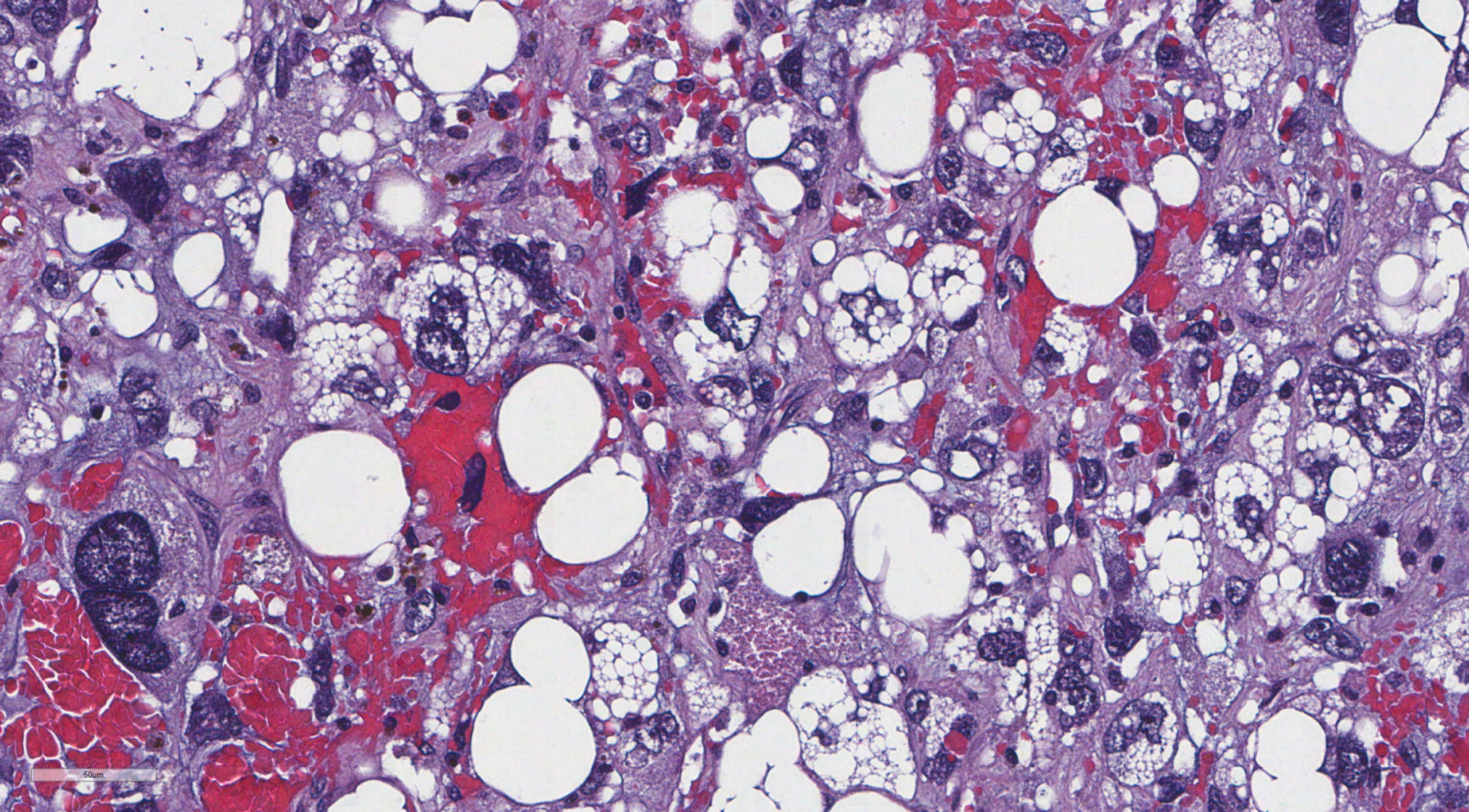

Supplement: figs2 [file mmcfigs2.jpg]
